# Enhancing Strength and Ductility of Rubberized Concrete Using Low-Cost Glass Jackets

**DOI:** 10.3390/polym18070841

**Published:** 2026-03-30

**Authors:** Panumas Saingam, Muhammad Noman, Burachat Chatveera, Gritsada Sua-Iam, Tahir Mehmood, Qudeer Hussain, Mohammad Alameri, Panuwat Joyklad

**Affiliations:** 1 Department of Civil Engineering, School of Engineering, King Mongkut’s Institute of Technology Ladkrabang, Bangkok 10520, Thailand; panumas.sa@kmitl.ac.th; 2 Civil and Coastal Engineering Department, University of Florida, Gainesville, FL 32603, USA; muhammadnoman@ufl.edu; 3 Department of Civil Engineering, Faculty of Engineering, Thammasat University, Rangsit Campus, Pathum Thani 12121, Thailand; cburacha@engr.tu.ac.th; 4 Department of Civil Engineering, Faculty of Engineering, Rajamangala University of Technology Phra Nakhon, Bangkok 10800, Thailand; gritsada.s@rmutp.ac.th; 5 Civil and Architecture Engineering Department, Sultan Qaboos University, Muscat 123, Oman; t.mehmood@squ.edu.om; 6 Department of Civil Engineering, Kasem Bundit University, Bangkok 10510, Thailand; ebbadat@gmail.com; 7 Civil Engineering Department, College of Engineering and Architecture, Umm Al-Qura University, Makkah 24382, Saudi Arabia; mdameri@uqu.edu.sa; 8 Department of Civil and Environmental Engineering, Faculty of Engineering, Srinakharinwirot University, Nakhonnayok 26120, Thailand; 9 Center of Excellence in Rail System Technology and Civil Engineering Material Innovation for Sustainable Infrastructure, Strategic Wisdom and Research Institute, Srinakharinwirot University, Bangkok 10110, Thailand

**Keywords:** rubberized concrete, glass chopped-strand mat (GCSM), axial compression, stress–strain behavior, sustainable construction materials

## Abstract

This study examines the compressive behavior and analytical modelling of natural and rubberized concretes (RuC) confined with low-cost glass chopped-strand mat (GCSM) jackets. A total of forty-two cylindrical specimens were tested under axial compression to assess the influence of rubber particle size, confinement configuration, and the number of GCSM layers. The RuC mixes were prepared by replacing 20% of fine aggregate by volume with crumb rubber of two size fractions: coarse (2.0 mm, retained on #10 sieve) and fine (0.425 mm, retained on #40 sieve). Both full- and strip-wrapping schemes were applied using two, four, and six layers of GCSM. The results demonstrated that GCSM jackets significantly enhanced the mechanical performance of both NAC and RuC specimens. Full wrapping provided the highest confinement efficiency, increasing compressive strength by up to 115% for NAC and 90% for RuC, while the ultimate axial strain increased by more than 1300% compared with unconfined specimens. Strip wrapping also improved performance, producing strength gains of 25–45% and strain increases of 250–500%. Analytical stress–strain models were developed through regression analysis, showing strong correlation with the experimental results (R^2^ = 0.80–0.99). The proposed GCSM jacket system demonstrates high potential as a sustainable and economical alternative for strengthening and retrofitting rubberized concretes, offering improved ductility and energy absorption while supporting circular material utilization. It is noted that the confinement ratio, size of rubberized aggregates, and their percentage replacement of rubberized aggregates should be consistent with the values used in this work in order to use the proposed analytical expressions.

## 1. Introduction

The construction industry is steadily moving toward materials and practices that lessen environmental impact while preserving structural reliability. Concrete, being the most widely used construction material, demands enormous quantities of raw aggregates and cement, which contribute substantially to global carbon emissions. At the same time, discarded vehicle tyres have become a major environmental concern, generating more than 1.5 billion end-of-life tyres every year and producing over 30 million tons of non-biodegradable rubber waste—around three percent of total landfill mass [1,2,3]. To address this issue, the reuse of crumb rubber (CR) as a substitute for natural fine aggregates has been studied as a sustainable approach that converts a persistent waste into a valuable ingredient for concrete [4,5,6]. Rubberized concrete (RuC) shows better impact resistance, toughness, and deformation capacity than normal concrete, yet this improvement in ductility comes at the expense of compressive strength and stiffness [7,8]. Several studies have carried out numerous possible remedies, such as surface treatment of rubber particles, blending with supplementary cementitious materials, and hybrid fiber reinforcement systems [9,10,11]. Despite these efforts, achieving a satisfactory balance between sustainability and mechanical performance remains a critical research challenge.

Fiber-reinforced polymer (FRP) jackets have proved a suitable and effective option to overcome the reduction in strength and, in many cases, surpass the mechanical capacity of NAC [12,13]. Previous studies have proved that an FRP jacket can improve compressive strength by about 80% to 150% and ultimate strain by 200% to 400%, depending on the stiffness of the confining system [14,15]. This improvement is possible because the FRP jacket limits the lateral dilation and delays brittle cracking, resulting in higher strength, improved ductility, and a more gradual, energy-dissipating stress–strain curve [2,16]. Over the past two decades, numerous studies have confirmed the benefits of carbon, glass, and basalt fiber composites in increasing the axial load capacity and deformation resistance of both circular and rectangular columns subjected to different loading conditions [17,18,19]. However, the relatively high cost and embodied energy of woven FRP fabrics continue to restrict their widespread use, particularly in areas with limited financial and material resources.

To overcome cost limitations, glass chopped-strand mat (GCSM) has emerged as a practical alternative. This material is composed of short glass fibers arranged randomly and bound with a light resin-compatible binder, providing nearly isotropic in-plane strength when used in multiple layers [20,21,22]. Its low cost and widespread availability make it a suitable alternative for large-scale retrofitting and strengthening work, especially in developing countries where cost constraints restrict the use of high-performance FRPs [23,24]. Previous studies have proved that a GCSM jacket can effectively enhance the load-carrying capacity and ductility of concrete elements, leading to measurable gains in both strength and energy absorption, even under modest confinement ratios [22]. The overall performance of GCSM, however, depends on several parameters such as the number of layers, resin saturation, and coverage pattern (full or strip). These parameters collectively determine the effective hoop stiffness and the confinement pressure transferred to the concrete [25]. Overall, GCSM offers a promising route to achieving mechanical enhancement with improved economic and environmental sustainability.

Recent research trends have also emphasized integrating low-cost confinement systems with sustainable concretes by replacing natural aggregates with recycled materials, such as crushed brick, ceramic waste, and rubber particles [26,27,28]. The usage of recycled materials and confinement yields dual benefits, as it reduces the consumption of virgin aggregates, assists in waste management, and extends the service life of existing structures. However, the behavior of confined RuC differs from NAC because of its higher lateral deformability, lower stiffness, and heterogeneous internal structure [29,30]. The lateral dilation of RuC under compression is typically greater than NAC, which affects how confinement pressure develops and consequently influences the efficiency of FRP jackets [14,31]. This interaction depends on the stiffness and configuration of the confinement as well as on the size and distribution of rubber particles, which control packing density, void ratio, and local stress transfer within the matrix [32,33]. Despite its practical importance, limited attention has been paid to the combined effects of rubber particle size and confinement parameters on the overall stress–strain response of RuC. Most of the existing studies on FRP-confined concretes have focused on high-strength, high-cost woven or unidirectional fiber composites, while research on cost-effective GCSM systems for confining RuC or other sustainable concretes remains scarce. Similarly, the performance of strip-wrapping configurations, which can offer material savings and easier application compared with continuous wrapping, has not been fully examined for GCSM, particularly in RuC systems [34,35]. The effectiveness of strip confinement, as well as its sensitivity to layer count, requires detailed investigation. Moreover, the influence of rubber particle size on confinement efficiency, stress transfer, and strain enhancement remains an open question, as particle size affects both the unconfined strength and the dilation behavior of RuC.

Although several studies have investigated the confinement of rubberized concrete using FRP composites such as CFRP and GFRP, the majority of these investigations have focused primarily on conventional FRP laminates applied in full-wrapping configurations. These systems are often effective in enhancing compressive strength and ductility; however, their relatively high cost and installation requirements may limit their widespread application. In contrast, GCSM composites represent a potentially more economical and adaptable reinforcement material, yet their application for the confinement of rubberized concrete has received limited attention in the literature. Furthermore, existing studies have rarely examined the effectiveness of partial confinement systems such as strip-wrapping configurations, which may provide a more cost-efficient strengthening solution while still improving structural performance. Therefore, the present study aims to address this research gap by experimentally investigating the compressive behavior of both normal aggregate concrete and rubberized concrete confined with GCSM composites using full- and strip-wrapping configurations. Additionally, analytical models are developed to predict the stress–strain behavior of the confined specimens, thereby contributing to a better understanding of the confinement mechanism and the practical applicability of this strengthening technique.

To bridge these gaps, the present work examines the compressive behavior of RuC containing 20% CR as a fine-aggregate replacement. The research examines the influence of particle size, confinement configuration (full and strip wrapping), and the number of GCSM layers (two, four, and six) on the strength and strain behavior of confined concrete. A total of 42 cylindrical specimens were tested under axial compression to evaluate confinement efficiency and strain enhancement mechanisms. In addition, regression-based analytical models were developed to describe the stress–strain behavior of both NAC and RuC under GCSM jacketing. The findings are expected to contribute to the development of low-cost, sustainable, and high-performance strengthening solutions for structural concrete incorporating recycled rubber.

## 2. Experimental Program

### 2.1. Test Matrix

The experimental work was organized into three main groups, as presented in Table 1, to evaluate the influence of rubber content, particle size, and the type of GCSM jacket. Group 1 (R0) consisted of conventional concrete without any rubber content. Group 2 (R40) comprised mixes with 20% CR replacing fine aggregate by volume, using particles retained on the #40 sieve (0.425 mm), whereas Group 3 (R10) used the same replacement level but with larger rubber particles retained on the #10 sieve (2.0 mm). All test specimens were cylindrical, measuring 150 mm in diameter and 300 mm in height. For each configuration, two companion specimens were prepared and tested to verify the consistency of the results, giving a total of 42 cylinders across all groups. The specimen identification system encodes both the concrete mix and the strengthening configuration. The prefixes R0, R40, and R10 denote the mix type described above. The suffix –Con indicates an unconfined control specimen. Suffixes beginning with F refer to full wrapping with GCSM, while those beginning with S refer to strip wrapping. The number between the letter and L indicates the number of GCSM layers.

### 2.2. Materials

**Figure 1:**
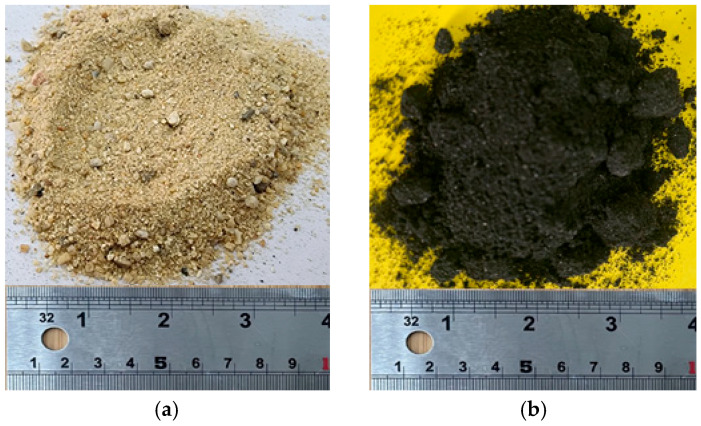

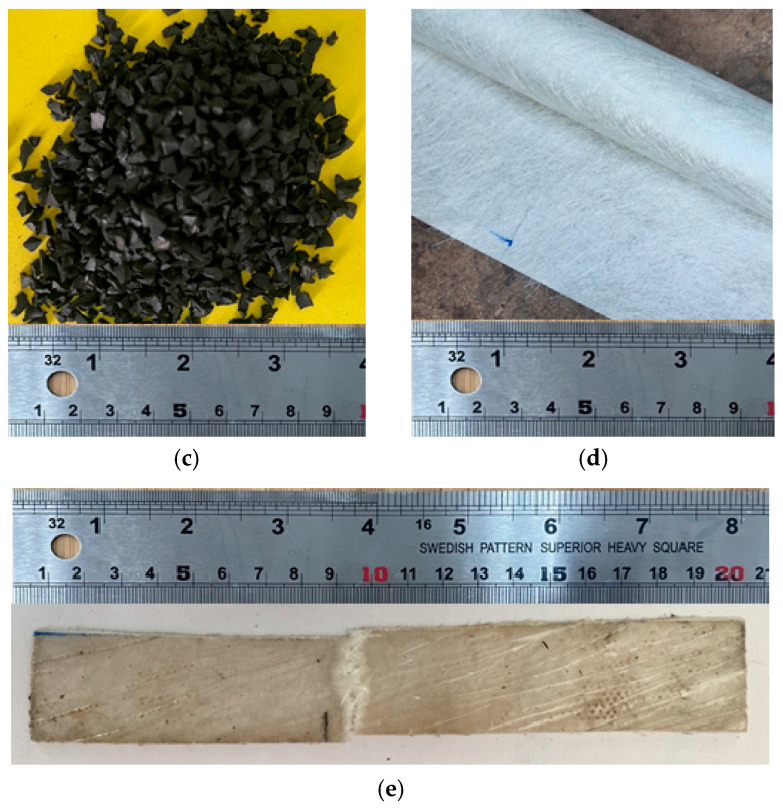
Fine materials used in this study: (**a**) natural sand; (**b**) fine rubber (40-mesh, 0.425 mm); (**c**) coarse rubber (10-mesh, 2.0 mm); (**d**) low-cost GCSM sheets used for external confinement; and (**e**) GCSM strip.

Ordinary Portland cement (OPC) conforming to ASTM C150 Type I [36] specifications was used as the primary binder. The cement had a specific gravity of 3.17 and a Blaine fineness of about 340 m^2^/kg. Natural river sand served as the fine aggregate, characterized by a fineness modulus of 2.6, specific gravity of 2.68, and water absorption of 1.6%. Crushed natural aggregate with a nominal maximum nominal size of 19 mm, a specific gravity of 2.76, and water absorption of 1.9% was used as the coarse fraction. To improve sustainability, CR was incorporated as 20% by-volume replacement of fine aggregate. Two CR gradations were selected: particles retained on the 40-mesh sieve (0.425 mm) and particles retained on the 10-mesh sieve (2 mm). These are referred to hereafter as fine and coarse rubber, respectively, as illustrated in Figure 1. The finer rubber fraction consisted mainly of powder-like particles smaller than 0.5 mm, whereas the coarser fraction contained irregular granules around 2 mm in size. The use of both sizes was intended to examine how particle fineness influences packing density, interfacial transition zone (ITZ) behavior, and the overall efficiency of external confinement, since previous studies have shown that smaller particles tend to increase matrix porosity while larger particles can introduce local stress concentrations [37,38]. The external confinement system consisted of GCSM sheets, a cost-efficient composite made of randomly oriented E-glass fibers bonded by a compatible resin binder. The random fiber orientation provides nearly isotropic in-plane properties, enabling uniform confinement around the cylinder surface. The mats were supplied in rolls 1.0 m wide and 0.5 mm thick, allowing for easy cutting and handling as shown in Figure 1d. Tensile properties determined in accordance with ASTM D3039M-08 [39] showed an average ultimate tensile strength of 180 MPa, a modulus of elasticity of 7470 MPa, and a rupture strain of 1.5%, confirming adequate stiffness and strength for confining applications. The tensile properties of GCSM composites were assessed by testing five standard-sized tensile coupons measuring 200 mm in length and 20 mm in width using a universal testing machine. The stress–strain behavior of the GCSM composite tensile strips exhibited a linear response up to the point of ultimate rupture. Most of the tensile coupons failed due to rupturing approximately in the middle, as shown in Figure 1e. In the past, many researchers have proposed the use of large-rupture-strain polyethylene terephthalate composites [40,41,42]. However, these are not easily available in many countries. The proposed GCSMs are low-cost and easily available. A two-part epoxy adhesive was used to bond the GCSMs to the concrete surface. The resin and hardener were mixed in a 2:1 ratio, providing the desired balance between workability and bond strength. The epoxy was applied in thin successive coats to ensure full impregnation and prevent air voids. The concrete surface was roughened before wrapping to enhance mechanical interlock and improve overall bond performance between the epoxy and substrate.

### 2.3. Specimen Preparation and Strengthening Procedure

**Figure 2:**
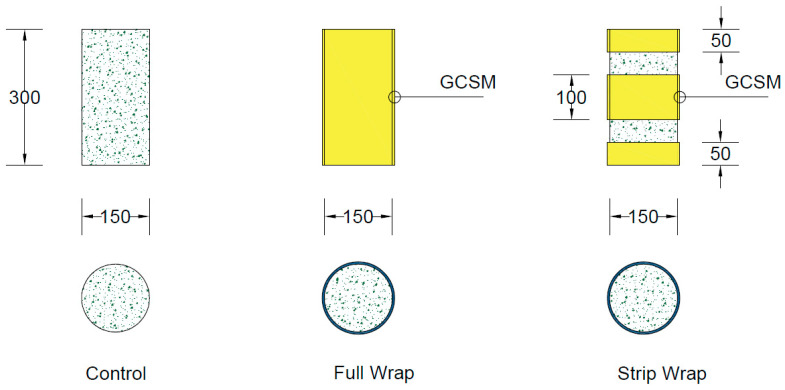
Geometry and strengthening details of concrete cylinders (dimensions in mm).

All concrete specimens were produced using the mix proportions described earlier, targeting a 28-day compressive strength in the range of 20–30 MPa. The mix design used for the concrete was as follows: 350 kg/m^3^ of cement, 700 kg/m^3^ of sand, and 1350 kg/m^3^ of aggregates, with a water-to-cement ratio of 0.8. All ingredients were mixed in a tilting-drum mixer to ensure uniform dispersion. The mixed ingredients were poured into cylindrical steel molds in three layers, with each layer compacted on a vibrating table to remove entrapped air and to ensure consistent density. After casting, the specimens were covered with plastic sheets to prevent moisture loss and left to set for 24 h before removing them from the molds. Subsequently, all cylinders were submerged in a curing tank containing clean water and kept at room temperature for 28 days to allow for complete hydration and strength gain. After curing, the surfaces were dried and prepared for the application of the GCSM jackets. Figure 2 shows the strengthening configurations.

**Figure 3:**
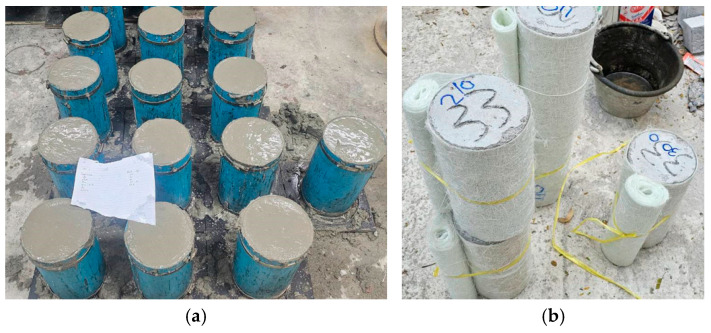

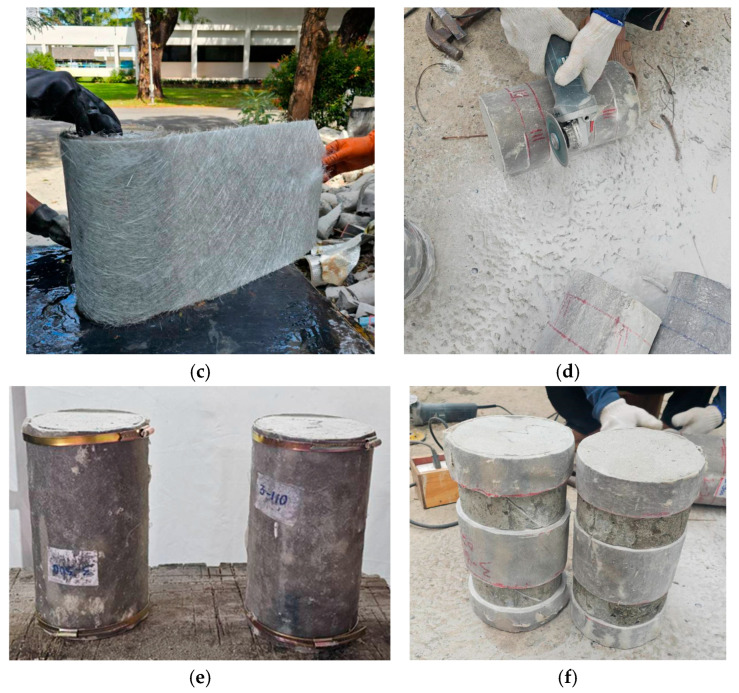
Overview of the specimen preparation and GCSM jacketing process: (**a**) construction; (**b**) dry wrap; (**c**) resin-impregnated wrapping; (**d**) strip cutting; (**e**) fully wrapped specimens; and (**f**) strip-wrapped specimens.

Prior to wrapping, the surface of each cylinder was thoroughly cleaned and lightly abraded with a mechanical grinder to improve texture and enhance the bond between the epoxy and the substrate. The epoxy adhesive, consisting of two components mixed in a 2:1 ratio of resin to hardener, was uniformly brushed over the marked wrapping zone. Pre-cut GCSM sheets, prepared with an overlap of about 100 mm, were then applied around the specimen using the hand lay-up method, as shown in Figure 3. A steel roller was used during and immediately after wrapping to press the mat firmly against the surface, eliminating trapped air and ensuring even contact. For specimens designed with multiple layers of GCSM, the same process was repeated, applying a new coat of epoxy between successive layers. Special attention was given to keeping the fiber alignment uniform and avoiding folds or misplacement during wrapping. Strip-wrapped specimens were prepared using 50 mm-wide GCSM bands spaced at 50 mm intervals along the cylinder height, as illustrated in Figure 2. After wrapping, all specimens were allowed to cure for 48 h under ambient conditions to ensure complete epoxy hardening before testing. The confined specimens were then labelled according to the identification system presented in Table 1 and stored in the laboratory until testing.

### 2.4. Instrumentation and Test Setup

**Figure 4:**
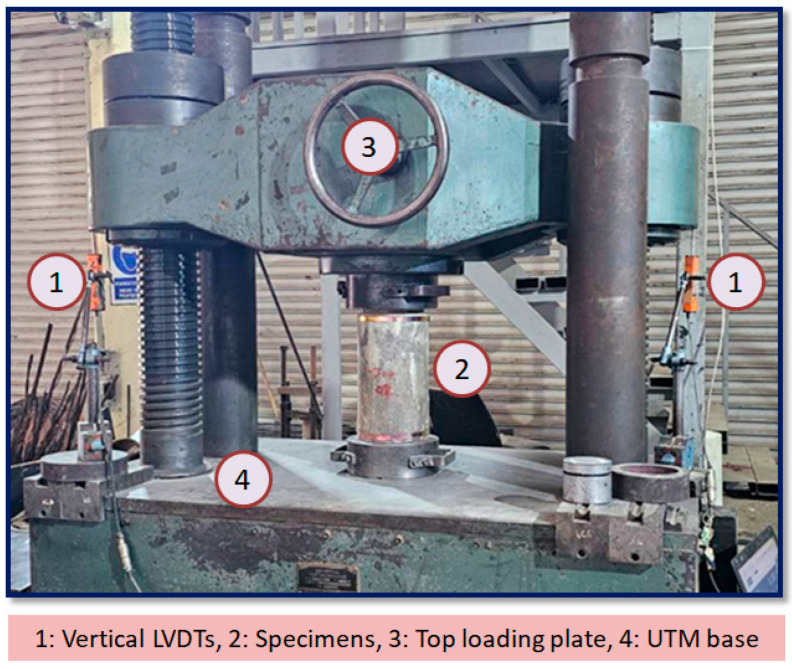
Instrumentation and loading arrangement for axial compression testing.

All compression tests were performed under monotonic axial loading using a servo-controlled universal testing machine (UTM) with a load capacity of 1000 kN, as shown in Figure 4. Each cylinder was positioned vertically between hardened-steel end plates to ensure uniform load transfer and minimize end restraint effects. Careful alignment of the specimen axis was carried out before loading to prevent eccentricity. Two linear variable displacement transducers (LVDTs) were mounted on a rigid steel frame to measure the deformation, as shown in Figure 4. A computerized data acquisition system was used to record the load and deformation values at set intervals. The experimental testing followed the standard ASTM C39 [43] procedure with minor modifications to accommodate the GCSM-confined specimens. A constant axial load rate of 0.5 MPa/s was applied. The compressive strength and ultimate axial strain were calculated from the load–displacement curves, and results were reported as the mean of three replicates per configuration. The experimental testing was performed under controlled laboratory conditions using a consistent, steady loading rate until specimen failure. The specimens were observed for the initiation and growth of cracks during the testing to record any instances of GCSM rupture or debonding. Stress–strain curves were prepared from the experimental results to evaluate the influence of confinement configuration and the number of GCSM layers on the compressive response of the RuC cylinders.

## 3. Results and Discussion

### 3.1. Failure Modes

**Figure 5:**
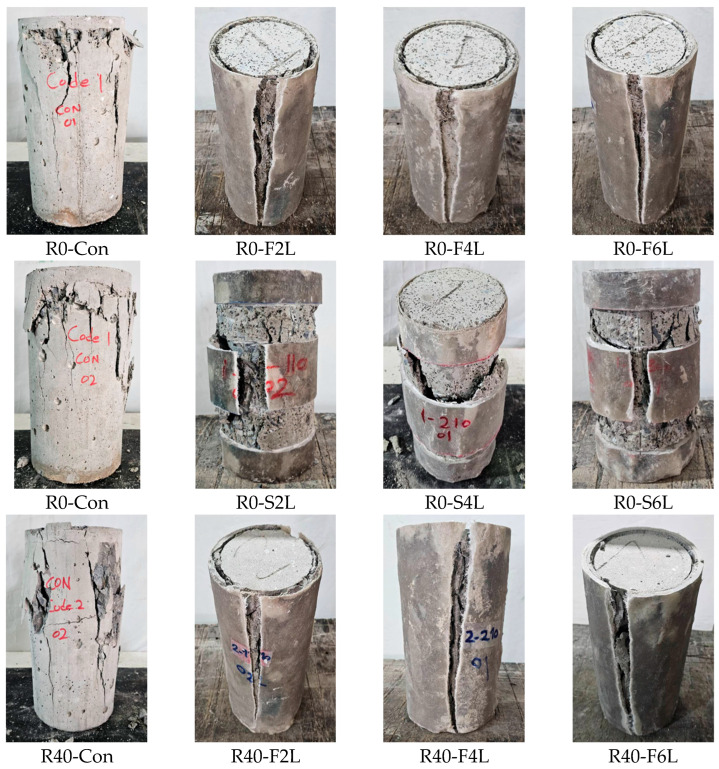

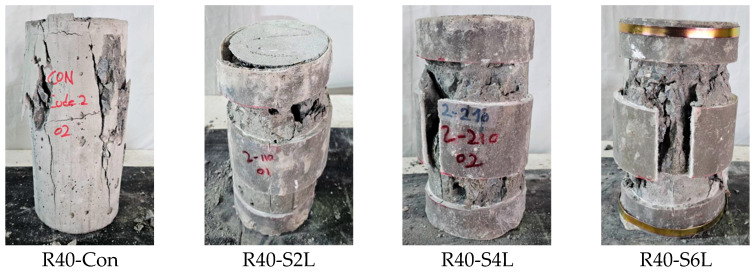
Typical failure modes of plain and GCSM-confined RuC cylinders under axial compression.

The observed failure modes of all specimens are shown in Figure 5. The control specimens of each group showed brittle splitting, marked by the development of vertical cracks followed by the abrupt spalling of concrete cover. These cracks were observed around the mid-height of the cylinder and quickly extended toward the ends, reflecting the absence of effective lateral restraint and limited ductility after peak load. Similar behavior of unconfined RuC has been reported in the literature [29,44], where it was observed that the inclusion of rubber increases crack width and reduces stiffness due to the weak rubber–matrix interface. For the GCSM-wrapped specimens, confinement markedly altered the failure pattern from brittle fracture to a more gradual and ductile mode. In full-wrapped cylinders (F2L–F6L), the outer GCSM jacket effectively restrained lateral dilation until rupture of the composite. Cracks were narrower and appeared primarily as fine vertical lines beneath the wrap, followed by progressive bulging of the middle zone. The specimens failed through localized crushing accompanied by GCSM rupture near mid-height, confirming that confinement pressure was fully mobilized before failure. Increasing the number of GCSM layers from 2 to 6 delayed rupture, reduced the extent of concrete spalling, and produced a more barrel-shaped profile at ultimate load. The enhanced ductility with thicker confinement layers can be attributed to the greater hoop stiffness and energy absorption capacity of the multi-layered GCSM composite in line with the previous findings [22,45]. In contrast, the strip-wrapped specimens showed partial confinement, and their failure was mainly dominated by crushing and splitting between adjacent strips. The cracks appeared in the unconfined parts of the cylinders and propagated upward until the concrete between strips crushed and dislodged. The addition of rubber particles in concrete enhanced the material’s flexibility, delaying sudden failure. This was particularly evident in full-wrapped specimens, where the elastic nature of the rubber matrix permitted significantly higher strain before ultimate rupture. Conversely, mixes with coarse rubber (R10) demonstrated more pronounced bulging before failure as compared to finer rubber (R40), implying that larger particles contribute to increased lateral deformation under similar stress. While the GCSM strips offered only partial lateral support, the pattern of alternating confined and unconfined sections triggered earlier cracking and diminished overall confinement efficiency relative to the fully wrapped specimens. Increasing the number of strip layers led to a modest performance gain, yet these specimens still failed to match the ductility exhibited by the continuously wrapped cylinders. Overall, the findings demonstrate that the GCSM jacket successfully changed the brittle failure mode of concrete into a ductile and energy-absorbing response. Full wrapping provided the most uniform lateral restraint and the highest resistance to crack propagation, whereas strip wrapping, though cost-efficient, offered limited confinement in the unwrapped zones. These results corroborate earlier findings on low-cost FRP and GCSM systems [21,25,46], validating their suitability for sustainable and economically viable strengthening applications.

### 3.2. Effect of GCSM Jacket on Compressive Stress–Strain Behaviour

**Figure 6:**
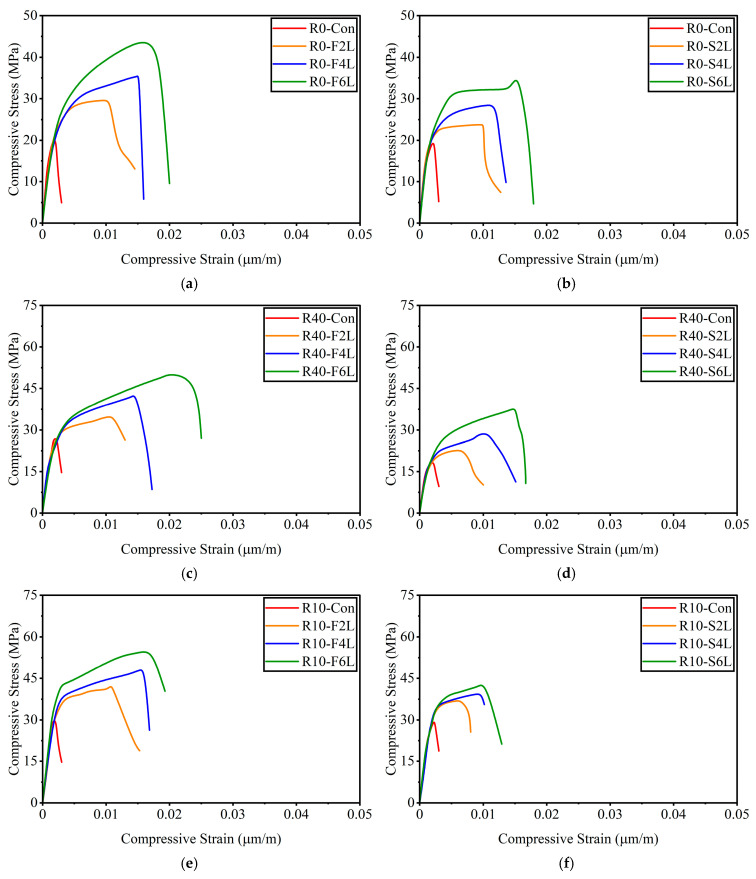
Compressive stress–strain response under axial loading: (**a**) Group 1 with full wrapping; (**b**) Group 1 with strip wrapping; (**c**) Group 2 with full wrapping; (**d**) Group 2 with strip wrapping; (**e**) Group 3 with full wrapping; (**f**) Group 3 with strip wrapping.

**Figure 7:**
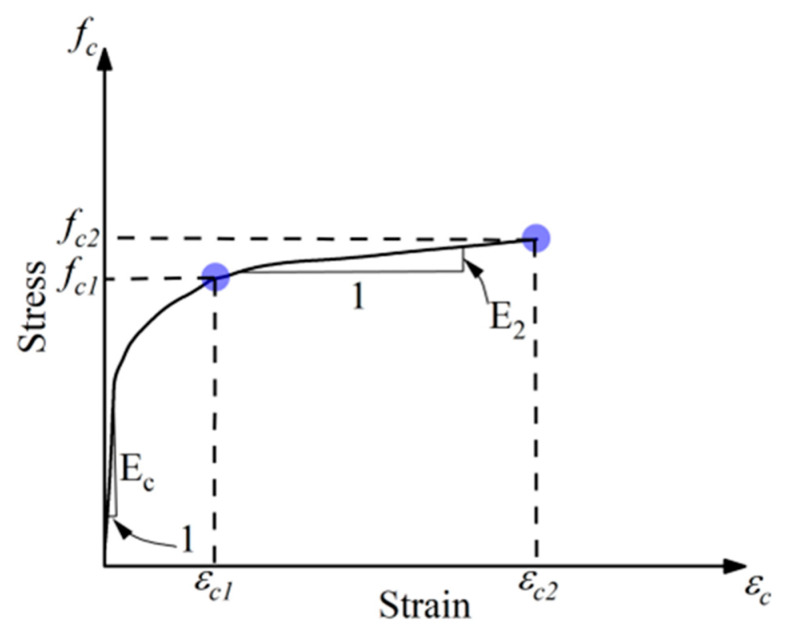
Idealized shape of compressive stress–strain curves of GCSM-confined concrete.

The curve consists of an initial linear portion governed by the elastic stiffness of unconfined concrete modulus $E_{c}$, followed by a nonlinear transition as lateral dilation begins and the confinement pressure from the GCSM jacket becomes active. Beyond the unconfined peak stress $f_{cl}$, the curve rises again to a confined peak stress $f_{c2}$ with a flatter second branch, representing the enhanced ductility and energy absorption typical of effectively confined concrete. The experimentally obtained stress–strain curves for all test groups are shown in Figure 6. The general shape of the compressive stress–strain response of GCSM-confined concrete is illustrated in Figure 7. The unconfined specimens (R0-Con, R40-Con, R10-Con) showed brittle failure and displayed a steep ascending branch and abrupt post-peak drop. GCSM-confined specimens exhibited a smoother stress–strain response and a significant rise in strain capacity, highlighting the positive impact of lateral confinement. Full-wrapped specimens showed a distinct bilinear behavior with a gradual post-peak decline, whereas the strip-wrapped specimens provided partial restraint that reduced cracking but caused a sharper loss of strength after reaching the peak. For the R0 mix, full wrapping increased the compressive strength from about 20 MPa to 44 MPa, as the number of layers increased from two to six, while the strip wrapping produced smaller yet noticeable gains. A comparable trend was observed in the RuC as well: the fine-rubber content mix achieved a strength of 51 MPa at six layers, whereas the coarse rubber mix achieved 52 MPa. These results confirm that strength and ductility improved consistently with additional GCSM layers, and the continuous jackets were more effective than the spaced strips.

Compared with conventional concrete, rubberized mixes showed higher ultimate strains but slightly reduced confinement efficiency per layer, particularly for coarse-rubber specimens, due to greater lateral expansion. Overall, the GCSM jacket transformed the brittle response of unconfined concrete into a ductile mode, producing well-defined second-branch behavior and a gradual energy-absorbing failure profile similar to that reported for other low-cost FRP systems [21,47]. The stress–strain responses clearly demonstrate that the GCSM jacket converts the brittle compressive behavior of concrete into a more ductile and energy-absorbing mode. The extent of enhancement is governed mainly by the continuity of confinement, being greater in fully wrapped specimens than in strip-wrapped ones, and by the number of GCSM layers, in agreement with confinement mechanisms reported in earlier FRP studies [48,49]. The axial compression of GCSM-confined rubberized concrete induces lateral expansion of the concrete core, which is resisted by the surrounding GCSM layers. In full-wrap configurations, the continuous GCSM jacket produces uniform hoop stress around the specimen, resulting in triaxial stress states that increase both compressive strength and ultimate axial strain. In strip-wrap configurations, the partial confinement generates localized lateral restraint, producing stress redistribution within the concrete core that mitigates premature cracking while allowing for controlled lateral dilation.

### 3.3. Quantitative Evaluation of Strength and Strain Improvement

Table 2 summarizes the experimental ultimate compressive strength and ultimate strain for all specimens. In the case of unconfined specimens, the strength of rubberized concrete specimens was found to be higher than the normal concrete. In this study, fine-rubber aggregates were utilized to replace natural sand, allowing us to explore their impact on concrete performance. The use of fine rubber not only enhanced the workability of the mix but also contributed to improved bonding within the cement matrix. This replacement can help fill voids and minimize porosity, potentially leading to superior mechanical properties. This study suggest that, under a controlled water-to-cement ratio, the incorporation of fine-rubber aggregates can yield a concrete composition that performs better than conventional mixes, opening new avenues for sustainable and resilient construction materials. The slight differences in stress values for R0-Con, R40-Con, and R10-Con for full and strip groups in Table 2 result from variations in the mixing conditions during the casting process. Further, in the case of confinement, the results confirm that external confinement using GCSM significantly enhanced both load-carrying capacity and ductility compared with the unconfined controls. The gain in strength was proportional to the number of confinement layers and more pronounced in full-wrapped specimens than in those wrapped with discrete strips. For Group 1 specimens, the application of two, four, and six GCSM layers increased the compressive strength by approximately 34%, 74%, and 115%, respectively, relative to the control. Whereas the strength gain in RuC specimens is 89% for the R40 mix and 75% for the R10 mix for six-layered full wrapping, confirming that GCSM confinement can effectively restore and even exceed the strength reduction caused by CR addition. The corresponding increase in ultimate strain, ranging from 600% to 800%, reflects a pronounced transformation in behavior of concrete from brittle failure to a significantly more ductile response. These improvements agree with findings by Joyklad et al. [20] and Thansirichaisree et al. [24], who reported similar trends for GCSM confinement where performance scaled with confinement material stiffness and continuity. Strip-wrapped specimens exhibited lower confinement efficiency, producing strength gains of 25 to 45% and strain increases of 250 to 500%. The reduced effectiveness stems from unconfined zones that allow for premature cracking and stress localization, consistent with partial-wrap studies on FRP-confined columns [25,50]. Nevertheless, even limited confinement markedly improved strain capacity, confirming that GCSM strips offer a cost-efficient alternative where full wrapping is impractical. Overall, the results demonstrate that low-cost GCSM provides confinement effectiveness comparable to conventional woven FRP systems when properly layered, making it a promising option for sustainable structural strengthening of RuC. Rubber particles within the matrix, particularly fine particles, contribute to enhanced deformability due to their lower modulus compared with natural aggregates. Under axial load, these particles deform elastically, delaying microcrack coalescence and transferring additional lateral stress to the GCSM layers. Coarse rubber particles create localized zones of higher lateral expansion, enhancing confinement in those areas but slightly reducing peak stress. The combined effect of rubber particle deformability and the lateral restraint provided by GCSM layers explains the observed increases in ductility and energy absorption, as the stress is redistributed from highly stressed zones to less stressed regions, delaying failure.

### 3.4. Influence of Confinement Configuration and Number of GCSM Layers

**Figure 8:**
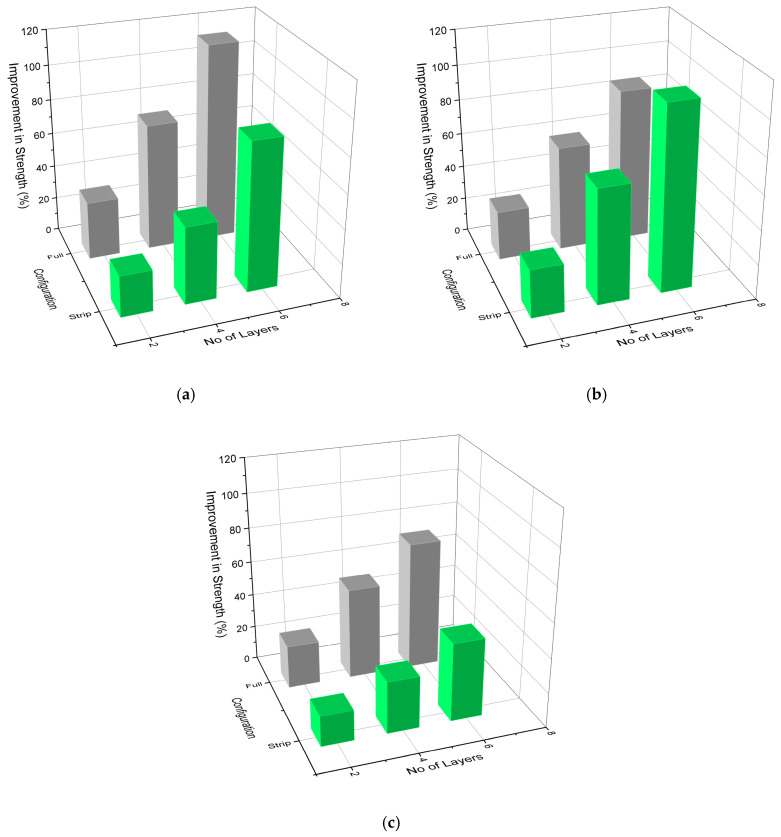
Influence of wrapping type and layer number on compressive strength enhancement of confined concrete: (**a**) Group 1—R0; (**b**) Group 2—R40; (**c**) Group 3—R10.

**Figure 9:**
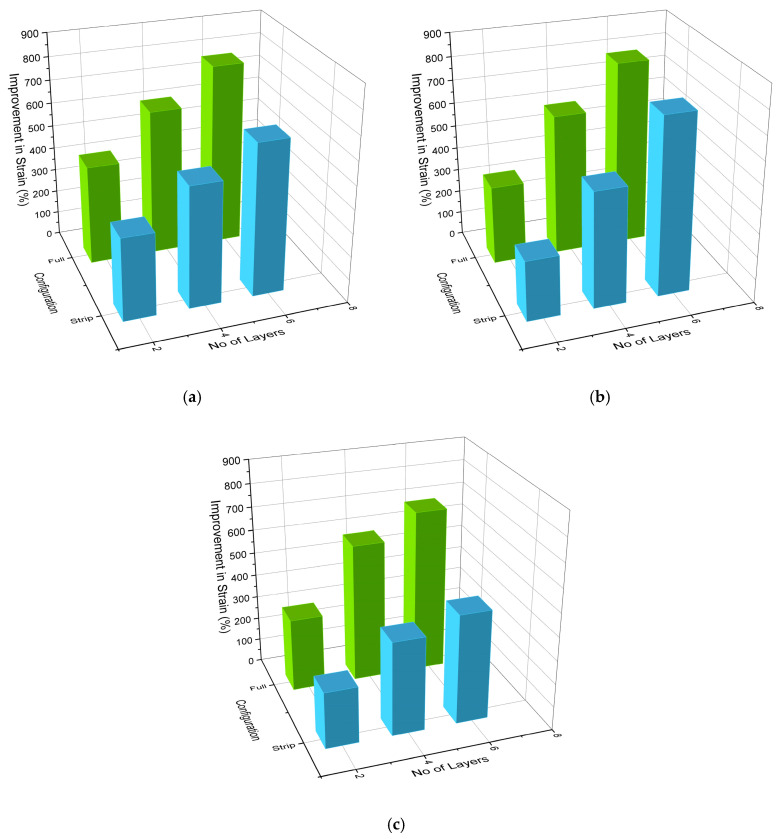
Effect of GCSM confinement configuration and layer number on strain capacity improvement: (**a**) Group 1—R0; (**b**) Group 2—R40; (**c**) Group 3—R10.

The influence of confinement type and number of GCSM layers on the mechanical performance of the specimens is illustrated in Figure 8 and Figure 9. The results show a consistent trend across all mixes, where full wrapping provided greater improvement in both ultimate strength and strain compared with strip wrapping, confirming the dominant role of confinement continuity. Strength gains ranged from approximately 25–35% for two-layer strip wrapping to over 100% for six-layer full wrapping, while strain enhancements followed a similar pattern, reaching up to 700–800% for the most heavily confined specimens.

This superior behaviour of fully wrapped specimens can be attributed to the continuous lateral pressure provided by the intact GCSM jacket, which delays crack propagation and suppresses premature spalling. In contrast, the unconfined gaps between strip bands allow for localized dilation and early cracking, reducing overall efficiency. Similar trends have been reported for partially wrapped FRP-confined columns by Zeng et al. [35] and Pham et al. [51], who observed that discontinuous confinement generates non-uniform stress distribution and limited post-peak ductility. The improvement in performance with increasing layer count is associated with the cumulative hoop stiffness and enhanced energy dissipation capacity of thicker GCSM jackets. Joyklad et al. [20] and Thansirichaisree et al. [24] noted comparable behaviour for chopped-strand composites, emphasizing that the confinement efficiency of non-woven FRP systems scales nearly linearly with the total fibre volume and wrap continuity. The present findings therefore validate the effectiveness of GCSM as a low-cost confinement material capable of delivering strength and ductility enhancements comparable to conventional woven FRP wraps.

### 3.5. Discussion

The strengthening behavior observed in the present study is consistent with trends reported in previous research on externally confined concrete using various composite reinforcement systems. Numerous studies have shown that confinement provided by FRP materials significantly enhances the compressive strength, ductility, and energy absorption capacity of concrete members due to the development of lateral confining pressure that delays crack propagation and restrains lateral dilation of concrete. For instance, research on CFRP- and GFRP-confined concrete cylinders has demonstrated substantial improvements in peak compressive strength and ultimate strain compared with unconfined specimens [52]. Similarly, investigations on textile-reinforced mortars and fiber-based confinement systems reported enhanced load-carrying capacity and improved post-peak behavior due to effective stress redistribution and crack-bridging mechanisms [53,54]. In recent years, natural fiber-based reinforcement materials have also attracted considerable attention due to their sustainability and cost-effectiveness. Studies utilizing hemp, jute, and other natural fibers for concrete confinement have reported significant increases in strength and deformation capacity while maintaining environmental benefits. The results obtained in the present study align with these findings, demonstrating that the proposed composite-strengthening approach effectively improves the mechanical response of the tested specimens by enhancing confinement efficiency and delaying failure. However, the magnitude of improvement depends on several parameters such as the reinforcement configuration, bonding characteristics, and mechanical properties of the composite material, indicating that the proposed system provides a competitive and sustainable alternative to conventional synthetic reinforcement techniques. The enhanced mechanical performance of GCSM-confined rubberized concrete can be explained by the interaction between the rubber particles and the confinement pressure. Rubber particles, due to their lower modulus and high deformability, allow for greater lateral dilation of the concrete matrix under axial load. This increased lateral expansion engages the surrounding GCSM layers more effectively, enhancing confinement pressure and delaying longitudinal crack propagation. Consequently, both compressive strength and axial strain capacity are improved. Differences between fine and coarse rubber particles were observed in terms of confinement efficiency and ductility. Fine-rubber particles distribute more uniformly within the cement matrix, promoting more homogeneous deformation and effective stress transfer under confinement. In contrast, coarse-rubber particles tend to form localized weak zones, resulting in increased lateral bulging but slightly lower peak stress. This explains why specimens with finer rubber exhibited higher strain enhancement with moderate strength improvement, while coarse-rubber mixes showed larger lateral dilation but marginally reduced strength. The significant increase in ultimate axial strain for confined rubberized concrete specimens can be attributed to the combined effects of the low stiffness of rubber particles and the lateral confinement provided by GCSM layers. As the matrix deforms, the rubber inclusions accommodate part of the strain, delaying microcrack coalescence. Meanwhile, the external GCSM layers restrain lateral expansion, producing a triaxial stress state in the concrete core. This triaxial stress condition suppresses brittle failure, resulting in enhanced ductility and energy absorption compared with unconfined or partially confined specimens [55,56,57,58].

## 4. Analytical Modelling

To capture the axial stress–strain response of GCSM-confined concrete (with and without crumb rubber), we adopt a smooth one-branch law that is constrained by two physically meaningful boundary conditions: the initial tangent stiffness at the origin, and the peak point of the confined curve. The constitutive form expressed in Equation (1):

(1) $$f_{c}=f_{cc}\left[\frac{k\left(\frac{\epsilon_{c}}{\epsilon_{cc}}\right)}{k-1+{\left(\frac{\epsilon_{c}}{\epsilon_{cc}}\right)}^{k}}\right]\;for\;\epsilon_{c}\leq \epsilon_{cc}$$

where $f_{c}$ is the stress corresponding to any value of strain $\epsilon_{c}$, $f_{cc}$ is confined peak compressive strength, and $\epsilon_{cc}$ is the corresponding peak strain. The research demonstrated that the Popovics modeling approach, as illustrated in Equation (1), effectively represented the early portion of the stress–strain relationship for concrete specimens confined by GCSM wrapping. Equation (2) defines the curvature parameter k, which controls the configuration of the stress–strain curve’s rising portion.

(2) $$k=\frac{E_{c}}{E_{c}-\frac{f_{cc}}{\epsilon_{cc}}}$$

where $E_{c}$ denotes the elastic modulus for plain concrete, calculated using the formula $4700\sqrt{f_{co}}$ (MPa), where $f_{co}$ represents compressive strength of unconfined concrete. The parameter k dictates how the material shifts from its early linear-elastic behavior to the nonlinear strain-hardening stage, thus influencing the curvature characteristics of the rising segment. Higher values of k correspond to a sharper gradient and more rapid attainment of maximum strength, whereas lower values signify a slower, more progressive stress development as strain increases.

### 4.1. Analytical Modelling for Natural Aggregate Concrete

#### 4.1.1. Analytical Expression for Peak Strength

**Figure 10:**
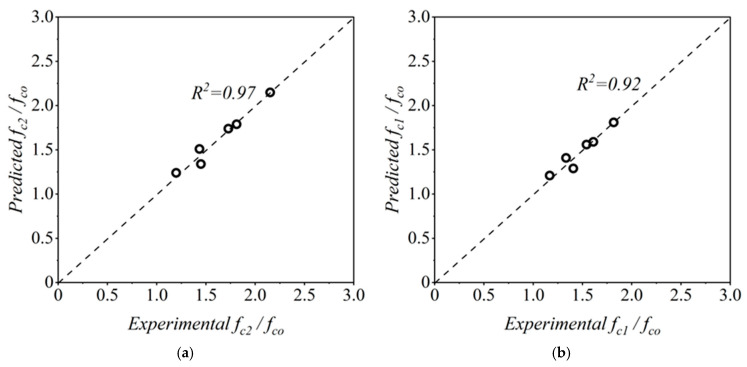

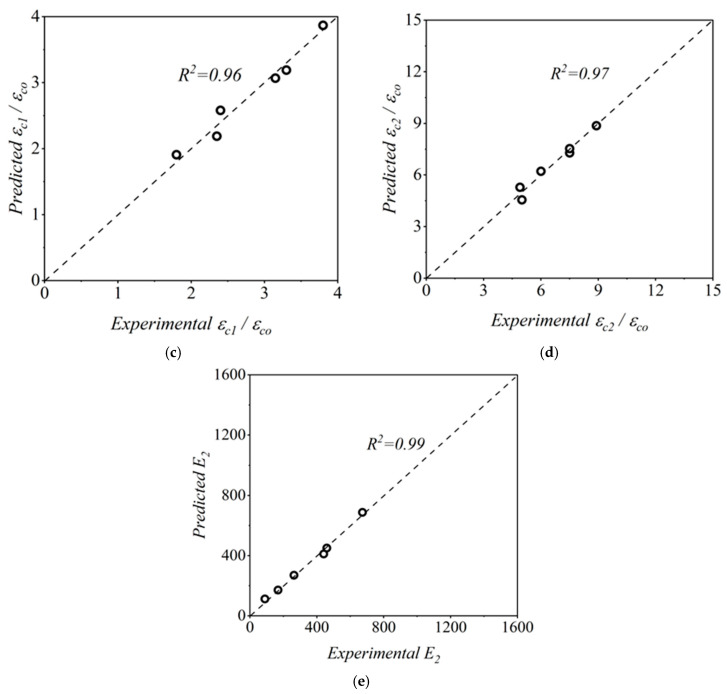
Comparison of experimental and predicted parameters for GCSM-confined NAC: (**a**) $f_{c2}/f_{co}$; (**b**) $f_{c1}/f_{co}$; (**c**) $\varepsilon_{c1}/\varepsilon_{co}$; (**d**) $\varepsilon_{c2}/\varepsilon_{co}$; and (**e**) $E_{2}$.

The peak compressive strength of GCSM-confined natural aggregate concrete (NAC) was correlated with the effective lateral confining pressure $f_{l}$. Regression analysis of the experimental data produced the best-fit model expressed in Equation (3):

(3) $$\frac{f_{c2}}{f_{co}}=1+3.621{\left(\frac{f_{l}}{f_{co}}\right)}^{1.10}$$

where $f_{c2}$ is the confined peak stress and $f_{co}$ is the unconfined concrete strength. The strong correlation coefficient of R^2^ = 0.97 confirms that the proposed model reliably captures the confinement effect of GCSM layers on compressive strength. The accuracy of the proposed expression is illustrated in Figure 10a, which shows a close alignment between predicted and experimental results across all confinement levels. The root mean square error (RMSE) was calculated to be 0.056.

#### 4.1.2. Analytical Expression for $f_{c1}$

Equation (4) provides the empirical formulation for $f_{c1}$, which characterizes the stress at the transition point on the rising curve segment of confined concrete, modeled in relation to the confinement ratio.

(4) $$\frac{f_{c1}}{f_{co}}=1+2.138{\left(\frac{f_{l}}{f_{co}}\right)}^{0.929}$$

The obtained correlation coefficient of R^2^ = 0.92 demonstrates a strong correlation between experimental and predicted values. The proposed equation effectively captures the pre-peak response, confirming its reliability for defining the transition stage of the confined specimens, as shown in Figure 10b. The root mean square error (RMSE) was calculated to be 0.058.

#### 4.1.3. Analytical Expression for $\epsilon_{c1}$ and $\epsilon_{c2}$

The strain parameters related to the transition stress ($\varepsilon_{c1}$) and the peak ($\varepsilon_{c2}$) stress are obtained through regression analysis. The developed expressions are given in Equations (5) and (6):

(5) $$\frac{\epsilon_{c1}}{\epsilon_{co}}=1+6.632{\left(\frac{f_{l}}{f_{co}}\right)}^{0.802}$$

(6) $$\frac{\epsilon_{c2}}{\epsilon_{co}}=1+14.028{\left(\frac{f_{l}}{f_{co}}\right)}^{0.553}$$

Both relationships produced excellent correlations ($R^{2}=0.96$ and $R^{2}=0.97$), confirming their robustness. Their RMSE values were 0.125 and 0.271, respectively. Figure 10c,d demonstrate that the analytical predictions follow the experimental trends closely, indicating that the proposed model effectively captures the strain enhancement produced by GCSM confinement.

#### 4.1.4. Analytical Expression for $E_{2}$

The post-peak modulus $E_{2}$, representing the slope of the descending branch of the stress–strain curve, was expressed as a function of both compressive strength and confinement ratio. The regression-based expression is presented in Equation (7):

(7) $$E_{2}=f_{co}^{-0.139}\times \left[1+3911.587{\left(\frac{f_{l}}{f_{co}}\right)}^{1.265}\right]$$

The model achieved a coefficient of determination $R^{2}=0.99$, signifying excellent agreement with experimental results. As shown in Figure 10e, the proposed relation effectively represents the confinement-induced stiffness variation and provides a reliable post-peak parameter for use in analytical stress–strain modeling of GCSM-confined concrete. The RMSE was calculated to be 17.20.

#### 4.1.5. Comparison of Predicted and Experimental Curves

**Figure 11:**
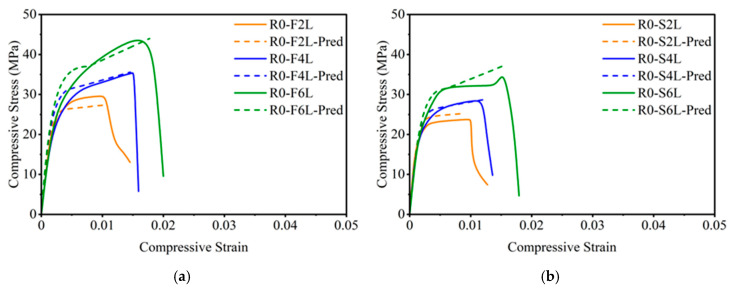
Comparison of experimental and predicted stress–strain curves for GCSM-confined NAC: (**a**) Group 1—full wrapping; (**b**) Group 1—strip wrapping.

A direct comparison between the analytical and experimental stress–strain curves for Group 1 specimens prepared from NAC is presented in Figure 11. The predicted responses closely replicate the experimental trends for both full- and strip-wrapping configurations. Minor deviations near the post-peak region are attributed to localized variations in fiber orientation and epoxy thickness. Overall, the model successfully represented both the strength and ductility enhancement achieved through GCSM confinement. These results are consistent with earlier studies on FRP-confined concretes [20,24,53], demonstrating that the simplified analytical framework accurately describes the confinement behavior of NAC.

### 4.2. Analytical Modelling for Rubberized Concrete

#### 4.2.1. Expression for Peak Strength for RuC

The confined compressive strength ($f_{c2}$) of GCSM-confined RuC was correlated with the confinement ratio $\left(f_{l}/f_{co}\right)$ through nonlinear regression. The best-fit model shown in Equation (8) describes the variation in strength enhancement as a function of lateral pressure:

(8) $$\frac{f_{c2}}{f_{co}}=1+3.874{\left(\frac{f_{l}}{f_{co}}\right)}^{1.083}$$

**Figure 12:**
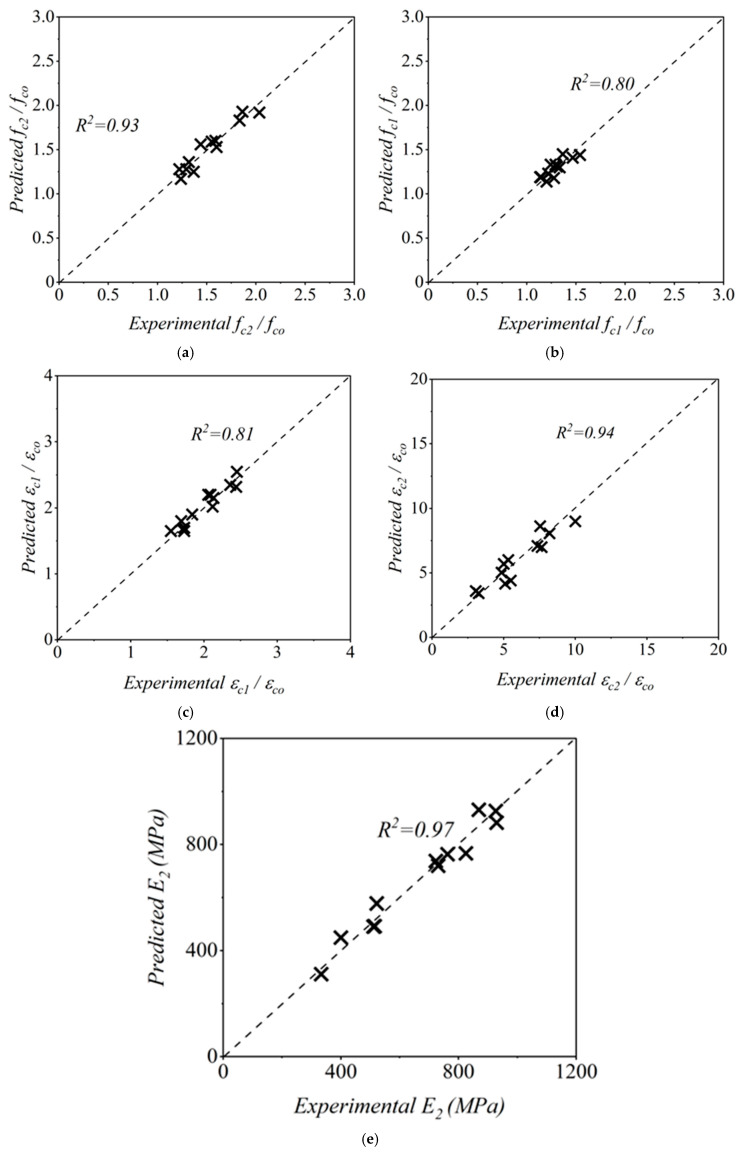
Comparison of experimental and predicted values for GCSM confined RuC: (**a**) $f_{c2}/f_{co}$; (**b**) $f_{c1}/f_{co}$; (**c**) $\varepsilon_{c1}/\varepsilon_{co}$; (**d**) $\varepsilon_{c2}/\varepsilon_{co}$; and (**e**) $E_{2}$.

The obtained correlation coefficient of $R^{2}=0.93$ indicates strong and consistent agreement between the analytical predictions and the experimental outcomes, despite the increased scatter caused by the non-uniform distribution of rubber particles. Even with this variability, the proposed model successfully reflects the overall improvement trend in compressive strength achieved through GCSM confinement. The comparison of measured and predicted results is presented in Figure 12a. The RMSE was calculated to be 0.0715.

#### 4.2.2. Expression for $f_{c1}$ for RuC

The stress corresponding to the transition point ($f_{c1}$) in the ascending region of the stress–strain curve was expressed as a function of the confinement ratio, as given in Equation (9):

(9) $$\frac{f_{c1}}{f_{co}}=1+1.216{\left(\frac{f_{l}}{f_{co}}\right)}^{0.759}$$

An $R^{2}$ value of 0.80 demonstrates that the model provides reliable estimation of the pre-peak stress behavior of confined RuC, even with the added deformability induced by rubber aggregates. The close alignment between predicted and experimental values, shown in Figure 12b, validates the model’s suitability for characterizing the initial confinement phase. The RMSE was calculated to be 0.064.

#### 4.2.3. Expression for $\epsilon_{c1}$ and $\epsilon_{c2}$ for RuC

The strain parameters corresponding to the transition stress $\epsilon_{c1}$ and the peak stress $\epsilon_{c2}$ were correlated with the confinement ratio using regression analysis, leading to the expressions given in Equations (10) and (11):

(10) $$\frac{\epsilon_{c1}}{\epsilon_{co}}=1+3.278{\left(\frac{f_{l}}{f_{co}}\right)}^{0.618}$$

(11) $$\frac{\epsilon_{c2}}{\epsilon_{co}}=1+20.177{\left(\frac{f_{l}}{f_{co}}\right)}^{0.734}$$

**Figure 13:**
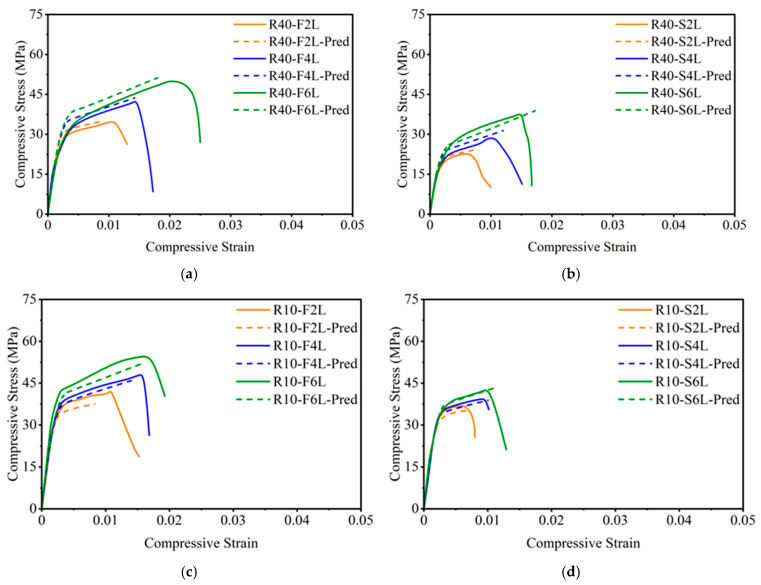
Comparison of experimental and predicted stress–strain curves for GCSM confined RuC: (**a**) Group 2—full wrapping; (**b**) Group 2—strip wrapping; (**c**) Group 3—full wrapping; (**d**) Group 3—strip wrapping.

The obtained correlation coefficients ($R^{2}=0.81$ and $R^{2}=0.94$) indicate strong predictive reliability. Their RMSE values were 0.174 and 1.049, respectively. These results confirm that the analytical model captures the enhanced deformability and strain capacity of GCSM-confined RuC. The higher exponents compared to NAC emphasize a stronger influence of confinement pressure on strain enhancement. The accuracy of the proposed model is illustrated in Figure 12c and Figure 13d.

#### 4.2.4. Expression for $E_{2}$ for RuC

The post-peak modulus ($E_{2}$), representing the stiffness degradation phase, was modeled as a function of both the confinement ratio and unconfined strength. The developed empirical relation is presented in Equation (12):

(12) $$E_{2}=f_{co}^{-0.019}\times \left[-9583.445+11155.006{\left(\frac{f_{l}}{f_{co}}\right)}^{0.041}\right]$$

The high correlation coefficient of $R^{2}=0.97$ demonstrates that the model effectively describes the stiffness evolution of confined RuC. The negative intercept reflects the reduced stiffness of rubberized mixes at low confinement pressures, while the increasing trend with higher confinement ratios confirms the recovery of stiffness through GCSM confinement. The predictive accuracy is shown in Figure 12e. The RMSE was calculated to be 44.896.

#### 4.2.5. Comparison of Predicted and Experimental Curves for RuC

The reliability of the proposed analytical model for GCSM-confined RuC was validated by comparing predicted and experimental data for all key parameters, as shown in Figure 12. Most of the data points lie close to the 45° reference line, with $R^{2}$ values ranging from 0.80 to 0.97, confirming the reliable predictive accuracy of the model for both strength and strain parameters. A further comparison of the complete stress–strain curves, shown in Figure 13, demonstrates that the model closely replicates the overall behavior of GCSM-confined RuC. For both fine-rubber (R40) and coarse-rubber (R10) mixes, the analytical curves accurately capture the nonlinear ascending portion and the gradual softening beyond the peak. Slight discrepancies in the descending region are mainly due to uneven rubber particle distribution, which affects the uniformity of confinement mobilization. In general, the regression-based models proposed in this research offer an accurate and realistic depiction of the confinement behavior of GCSM-encased RuC. The strong agreement between the experimental data and the predicted responses, as shown in Figure 12 and Figure 13, demonstrates the reliability and suitability for practical use in assessing and designing sustainable FRP-confined RuC systems.

## 5. Practical Implementation of GCSM Confinement

The experimental results indicate that GCSM composites can be effectively used to strengthen existing concrete members or enhance the performance of rubberized concrete in new construction. Full wrapping provides maximum confinement and is recommended for members requiring significant ductility and strength enhancement, while strip wrapping offers a cost-effective alternative where partial confinement is sufficient.

For practical applications, the number of GCSM layers should be selected based on the desired strength and strain enhancement, considering the limitations of handling and bonding. The adhesive should be applied uniformly to ensure good contact between the GCSM layers and the concrete surface, and adequate curing (e.g., 24 h at room temperature) is recommended to achieve full polymerization. The technique is particularly suitable for low-cost, sustainable construction or retrofitting projects where conventional FRP systems may be prohibitively expensive.

While these recommendations are based on experimental observations, further research is encouraged to develop design guidelines and incorporate GCSM confinement into standardized structural design procedures.

## 6. Conclusions

This study focused on the effects of incorporating rubber content as a partial replacement of fine aggregates, the influence of different GCSM confinement layers, and configurations in the form of full and strip wrapping. The conclusions drawn from the experimental and analytical evaluation of GCSM-confined NAC and RuC specimens are as follows:

- GCSM wrapping effectively transformed the brittle failure of concrete into a more gradual and ductile mode characterized by fine vertical cracking and localized bulging instead of abrupt spalling.
- Full GCSM wrapping provided the highest confinement efficiency, increasing compressive strength by up to 115% for NAC specimens and 90% for RuC specimens compared with unconfined concrete. In addition, the ultimate axial strain increased by more than 1300%, demonstrating a significant improvement in deformation capacity.
- Strip wrapping also improved the mechanical performance but with lower efficiency, producing compressive strength gains ranging from 25% to 45% and strain improvements between 250% and 500%, while offering a more economical solution due to reduced material usage.
- RuC mixes showed higher deformability and confinement sensitivity as compared to NAC. Fine-rubber mixes (R40) achieved superior strain enhancement due to improved lateral ductility, whereas coarse rubber (R10) exhibited greater lateral expansion but slightly lower confinement efficiency.
- Increasing the number of GCSM layers consistently improved both compressive strength and ductility due to higher hoop stiffness and delayed rupture of the composite jacket.
- The regression-based models developed for both NAC and RuC accurately predicted the stress–strain response, including transition and peak stresses, strain enhancement, and post-peak modulus, with excellent correlation (R^2^ = 0.80–0.99).
- Overall, GCSM confinement demonstrated strong potential as a cost-effective and sustainable strengthening technique, providing substantial strength improvements (up to 115%) and exceptional strain enhancement (exceeding 1300%), making it a promising alternative to conventional FRP systems for retrofitting rubberized concrete in resource-constrained regions.

## Figures and Tables

**Table 1:** Details of concrete specimens tested.

Group	Specimens ID	Rubber Content (%)	Rubber Size (Mesh)	Strengthening Configuration	Number of Layers
1	R0-Con	0	-	None	None
	R0-F2L	0	-	Full	2
	R0-F4L	0	-	Full	4
	RO-F6L	0	-	Full	6
	R0-S2L	0	-	Strip	2
	R0-S4L	0	-	Strip	4
	R0-S6L	0	-	Strip	6
2	R40-Con	20	40	None	None
	R40-F2L	20	40	Full	2
	R40-F4L	20	40	Full	4
	R40-F6L	20	40	Full	6
	R40-S2L	20	40	Strip	2
	R40-S4L	20	40	Strip	4
	R40-S6L	20	40	Strip	6
3	R10-Con	20	10	None	None
	R10-F2L	20	10	Full	2
	R10-F4L	20	10	Full	4
	R10-F6L	20	10	Full	6
	R10-S2L	20	10	Strip	2
	R10-S4L	20	10	Strip	4
	R10-S6L	20	10	Strip	6

**Table 2:** Ultimate strength and strain characteristics of specimens under GCSM confinement.

Group	Specimen ID	Stress (MPa)	Increase in Stress (%)	Ultimate Strain (mm)	Increase in Strain (%)
1	R0-Con	20.45	-	0.002	-
	R0-F2L	27.40	34	0.0106	429
	R0-F4L	35.58	74	0.0146	629
	R0-F6L	43.97	115	0.0177	787
	R0-Con	19.88	-	0.002	-
	R0-S2L	24.65	24	0.0091	356
	R0-S4L	28.81	44.91	0.0124	522
	R0-S6L	37.00	86.12	0.0151	654
2	R40-Con	27.08	-	0.002	-
	R40-F2L	34.89	28.83	0.0088	341
	R40-F4L	43.65	61.19	0.0142	610
	R40-F6L	51.21	89.12	0.018	800
	R40-Con	18.83	-	0.002	-
	R40-S2L	24.09	27.92	0.0072	260
	R40-S4L	31.36	66.53	0.012	500
	R40-S6L	38.84	106.29	0.01724	762
3	R10-Con	30.00	-	0.002	-
	R10-F2L	37.52	25.07	0.0083	316
	R10-F4L	45.91	53.04	0.014	600
	R10-F6L	52.39	74.63	0.0161	707
	R10-Con	29.89	-	0.002	-
	R10-S2L	35.18	17.69	0.0068	242
	R10-S4L	38.89	30.10	0.0101	403
	R10-S6L	43.48	45.47	0.0114	470

## Data Availability

The original contributions presented in this study are included in the article. Further inquiries can be directed to the corresponding author.
